# Metabolic profiling of dialysate at sensitized acupoints in knee osteoarthritis patients: A study protocol

**DOI:** 10.1097/MD.0000000000017843

**Published:** 2019-11-11

**Authors:** Sheng Li, Xiao Ning Chai, Chuan Yi Zuo, Peng Lv, Yong Tang, Hui Juan Tan, Li Zhou Liu, Hai Yan Yin, Shu Guang Yu

**Affiliations:** a Chengdu University of Traditional Chinese Medicine, Chengdu; b Southwest Medical University, Luzhou, China; c Centre for Health, Activity and Rehabilitation Research, School of Physiotherapy, University of Otago, Dunedin, New Zealand.

**Keywords:** KOA, LS-MS/MS, micro dialysis, protocol, sensitized acupoints

## Abstract

**Background:**

Acupuncture therapy is frequently used to treat Knee Osteoarthritis (KOA) in clinic, and usually used local acupoints near the diseased knees as therapeutic targets. Some local acupoints appeared sensitization phenomenon which was called sensitized acupoints, which were regarded as important therapeutic targets to get better therapeutic effect on clinic. Therefore, it is necessary to explore the biological basis of acupoint sensitization. Meanwhile, there is a lack of an analysis of the metabolism for sensitized acupoints in KOA patients. Considering that acupuncture effect could be multi-targeted, omics (such as metabolomics) may be a useful method to reveal the relationship between sensitized acupoints and clinical efficacy of acupuncture.

**Methods and analysis:**

This study is a parallel design trial. Thirty KOA patients and 30 healthy volunteers will be recruited in this study. Mechanical pain threshold will be measured by Electron Von frey in order to confirm the highest sensitized acupoints. Then collect tissue fluid from the highest sensitized acupoints by micro dialysis technical, then apply electro-acupuncture method on the highest sensitized acupoints to treat KOA patients, after 20 sessions treatments, measure and collect again. Liquid chromatography-tandem mass spectrometry method will be used to analyze the metabonomics of dialysate.

**Results:**

This study will provide a high-quality evidence to reveal the local molecular mechanism of acupuncture sensitized acupoints for patient with KOA.

**Conclusion:**

This study will provide up-date evidence of whether acupuncture sensitized acupoints have local molecular mechanism for KOA. Trial registration number: NCT03599180 (24 Jul. 2018)

## 1 Introduction

Knee osteoarthritis (KOA) is a common type of arthritis seen in the middle-aged and elderly population^[1,2]^ which is accompanied with chronic pain, inflammation, and impaired motor function, leading to deterioration of quality of life.^[3]^ The main therapeutic goals in the treatment of osteoarthritis are pain relief and functional improvement.^[4]^ Related systematic review showed acupuncture is an effective therapy in treating KOA especially on the short-term pain relief and short and long-term physical function improve.^[5]^

Many studies chose acupoints around the knee joint to treat KOA.^[6–13]^ KOA has some clear pathological reaction points around the knee joint, even some of them overlap with acupoints.^[14–15]^ Some local acupoints appeared sensitization phenomenon, such as Zusanli (ST35), Yanglingquan(GB34), Sanyinjiao(SP6), and Dubi(ST35) were appeared heat-sensitized in KOA patients.^[16]^ Meanwhile, the improvement of joint pain, morning stiffness, joint swelling and walking ability after treatment at heat-sensitized acupoints with heat moxibustion method was much more apparent as compared with conventional moxibustion method.^[17]^ This sensitized phenomenon also existed in other diseases such as coronary artery disease,^[18]^ functional bowel disorders,^[19]^ cholecystitis,^[20]^ and it has been defined as acupoints sensitization which refers to the change of acupoints from silence to activation during pathological process.^[21]^

After acupoints sensitized, their physical properties may change, for instance, volt-ampere characteristics.^[22–24]^ And substances of acupoints may also change, such as ATP,^[25]^ adenosine,^[26]^ histamine (HA), serotonin (5-HT), calcitonin gene-related peptide (CGRP), substance P (SP), and TRPV-1 channel proteins.^[27–29]^ Those means the sensitized acupoints were caused by the change of a variety of substances or factors, not just one of the substances or factors. It is not known whether other substances work in sensitized acupoints. Most of these studies were conducted in animal models, it is necessary to conduct metabolomics studies in human body from a broad perspective on the matter of sensitized points, especially small molecules matter. Therefore, we designed this project to use micro dialysis, liquid chromatography-tandem mass spectrometry (LC-MS/MS) and high-throughput sequencing technology to analyze the non-targeted metabolomics, to screen out the key substances of sensitized acupoints.

## 2 Methods

### 2.1 Overview

This trial is a single-center, normal controlled, parallel group, clinical trial. The trial is an explorative, pilot trial designed to reveal the change of the main material group and key substances in sensitized acupoints caused by knee osteoarthritis.

The trial is being conducted at the affiliated hospital of Chengdu University of TCM, Acupuncture Outpatient Department, Si Chuan, and China. The trial was approved by the Chinese ethical committee on human research (reference NO. 2018KL-042), and this program is part of our clinical registration subject registered on www.clinical.trials.gov (reference NO. NCT03599180). The trial will be reported in compliance with the CONSORT statement (www.consort-statement.org).

### 2.2 Sample size calculation

This study aims to investigate basic information about sensitized acupoints of KOA patients, rather than to satisfy hypothesis testing. Hence, the sample size was estimated by based on a rationale about feasibility, precision about the mean and variance, regulatory considerations and ethical issues that prohibit over-recruitment of participants. Considering an estimated 20% dropout rate, we assured that the sample size exceeded the minimal number needed to ensure the validity of the mean, effect size and rationale of feasibility. Therefore, a sample size of 60 participants was estimated.

### 2.3 Recruitment KOA patient and healthy volunteers

**Figure 1:**
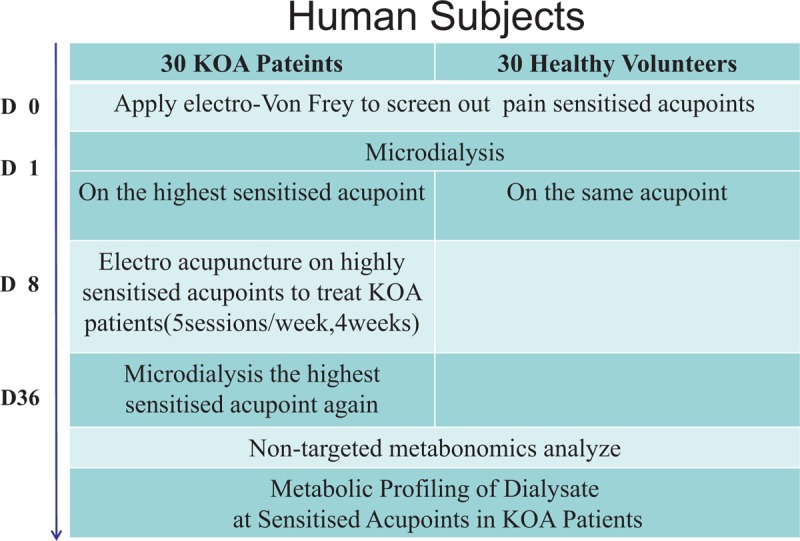
Illustrates the flow diagram of studies identified.

A total of 30 KOA patients and 30 normal volunteers will be included, flow diagram in Figure 1. No interim analysis will take place. Subjects enrolled will have some financial compensation, and KOA patients can also receive 4 weeks free electro-acupuncture treatments. And all information about the subject will be kept strictly confidential until the end of the trial.

#### 2.3.1 Diagnostic criteria of KOA patients

The criteria referenced the “*Osteoarthritis Diagnosis and Treatment Guideline*” (2018) formulated by Joint Surgery Group of Bone Science Branch of the Chinese Medical Association.

I. Repeated knee pain occurred in the past month.
II. X-ray imaging taken standing or weight-bearing, seen ipsilateral knee joint space narrowing, bone sclerosis or cystic changes with articular cartilage, knee edges seen with osteophyte formation.
III. Patients age ≥50 years.
IV. Morning stiffness is less than 30 minutes.
V. Bone crepitus or bone friction feeling during activity.

Meet the diagnostic criteria I+ (any 2 of II, III, IV, V) can diagnose knee osteoarthritis.

#### 2.3.2 KOA classification criterion

According to the imaging examination X-ray *Kellgren* and *Laerence* classification standards are as follows:

I level: Knee joint space is suspiciously narrowed, possibly with the formation of osteophytes.
II level: Knee joint space is suspiciously narrowed, visible osteophyte formation
III level: The narrowing of the joint space of the knee joint was relatively clear, showing that there was a moderate amount of osteophyte formation and may be accompanied by sclerotic changes.
IV level: It can be seen that there are a large number of osteophyte formations, and the joint space of the knee joint is significantly narrowed and may be accompanied by sclerotic lesions and obvious joint deformities.

KOA patients in- and exclusion criteria are listed in Table 1.

**Table 1:**
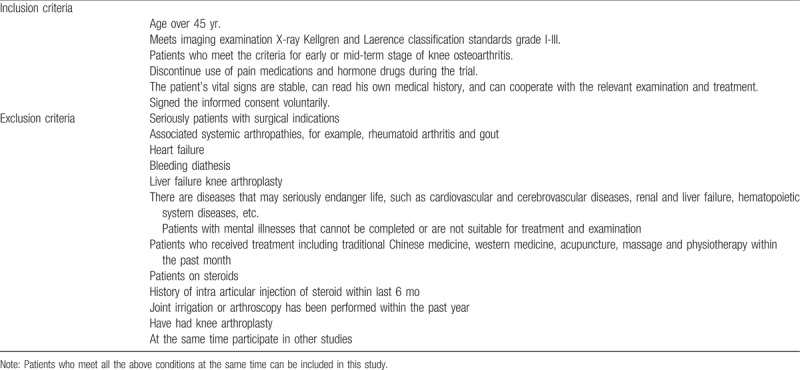
Inclusion and exclusion criteria of KOA patients.

#### 2.3.3 Recruitment volunteers

Thirty healthy volunteers whose age over 45-year will be recruited in control group.

### 2.4 Mechanical pain threshold

Mechanical pain threshold (MPTs) test will be performed according to the recommendations of the German Research Network on Neuropathic Pain.^[30]^ Electronic Von frey will be used to contact the acupoints around the knee osteoarthritis joint and the same acupoints of volunteers. The final threshold was the geometric mean of three series of ascending and descending stimulus intensities.

**Figure 2:**
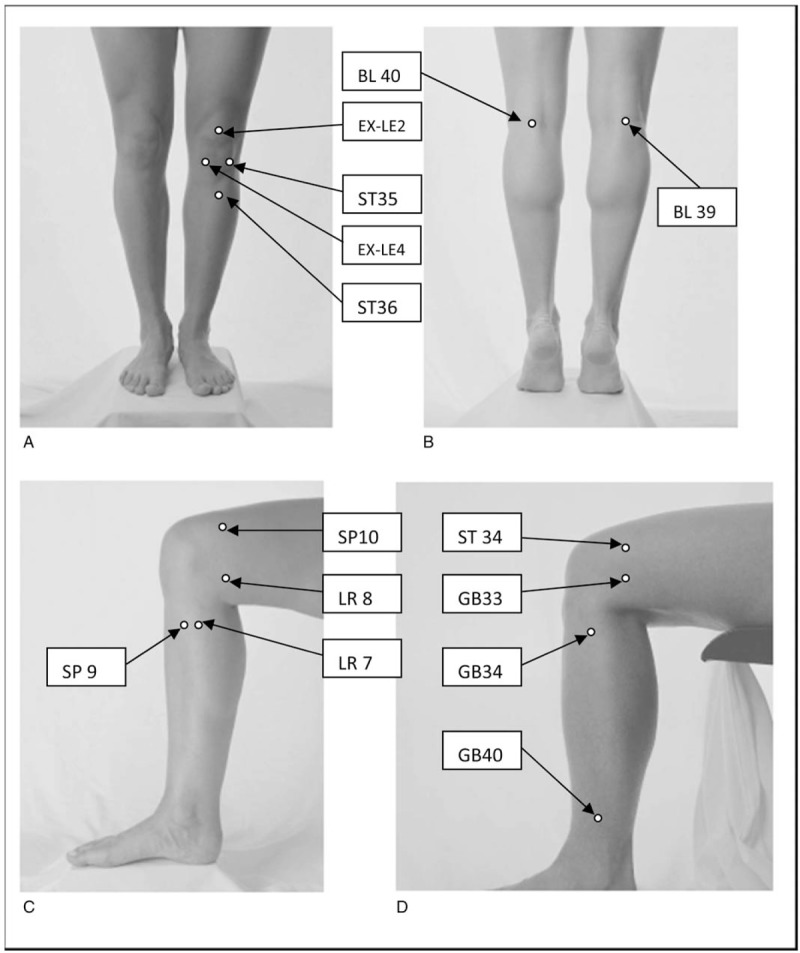
Illustrates these local points in knee osteoarthritis acupuncture therapy.

Acupoints choose based from the analysis of the acupoints selection rules for randomized controlled trials of acupuncture for knee osteoarthritis in the modern literatures.^[31]^ And the point's locations are showed in Figure 2.

### 2.5 Micro dialysis

The highest sensitized acupoint of KOA patients and the same acupoint of volunteers will choose for micro dialysis. Disinfect locally with 75% alcohol, cover disposable surgical hole towel. The skin overlying the muscle of the puncture sites will be anesthetized with a local injection (0.5 ml) of Lidocaine (Xylocaine 20 mg /ml), carefully avoiding anaesthetized the underlying muscle.

Insert introducer cannula into muscle tissue below acupoint. Guide the catheter (CMA 66 Liner Catheter, Membrane: PAES, Membrane length 10 mm, Cut-off 20,000 Dalton, Inlet 400 mm, Outlet 100 nm. M dialysis AB, Sweden) gently in to the sharp end of introducer cannula (Important: be very careful when entering membrane into introducer cannula). When catheter and membrane are in position, remove introducer cannula. Penetrate vial cap with a cannula (preferably 21G). Position cannula at the catheter outlet, removes the cannula from outlet, and then connect microvial. Then connect catheter to syringe filled with Perfusion Fluid (CMA, M dialysis AB, Sweden). Place the syringe in the microdialysis pump (CMA 106, M dialysis AB, Sweden). A 5 minutes flush will start indicated by a green signal every other second. Then after 120 minutes of equilibration the dialysate will collect every 60 minutes for a total of 3 collections. The dialysate will store frozen in –80° refrigerator as soon as possible.

### 2.6 Intervention method

After highly sensitized acupoint screening and dialysate sample collecting, 5 to 6 sensitized acupoints will be chose to the main points. Then, KOA patients will receive electro-acupuncture treatment 5 sessions per week for 4 weeks, with continuous wave and durations of 30 minutes, the electricity intensity is based on the patient's tolerance. Relevant concomitant care and interventions that are permitted or prohibited during the trial. At the end of the treatment, the sensitized acupoints will be detected again to see whether MPTs had changed, and the previous most sensitized acupoint will be collected dialysate sample again.

### 2.7 Liquid chromatography - tandem mass spectrometry (LC-MS/MS)

LC-MS/MS analysis will be performed using a Vanquish UHPLC system (Thermo Fisher) coupled with an Orbitrap Q Exactive HF-X mass spectrometer (Thermo Fisher) operating in the data-dependent acquisition (DDA) mode. Samples (dialysate) were injected onto an Accucore HILIC column (100 × 2.1 mm, 2.6 μm) using a 20-minute linear gradient at a flow rate of 0.3 mL/minute. The eluents of the positive polarity mode were eluent A (0.1% FA in 95% ACN, 10 mM ammonium acetate) and eluent B (0.1% FA in 95% ACN, 10 mM ammonium acetate). The eluents of the negative polarity mode were eluent A (95% ACN, 10 mM ammonium acetate, pH 9.0) and eluent B (50% ACN, 10 mM ammonium acetate, pH 9.0). The solvent gradient was set as follows: 2% B, 1 minute; 2% to 50% B, 16.5 minutes; 50% to 2% B, 2.5 minutes. Q-Exactive HF-X mass spectrometer was operated in positive/negative polarity mode with spray voltage of 3.2 kV, capillary temperature of 320°C, sheath gas flow rate of 35 arb and aux gas flow rate of 10 arb.

In order to detect metabolites, we will carry on the principal component (PCA) and orthogonal partial least-squares (OPLS-DA) analysis. Through MzCloud database the potential differences metabolites will be identified, then use Kyoto encyclopedia (KEGG) gene and genome data analyze the related metabolic pathways.

### 2.8 Follow up

One week after microdialysis, all patients will be contacted by phone to ensure they are safe.

### 2.9 Outcome measure

The primary outcome is the identified potential differences metabolites between KOA group and volunteer group will be the outcome measures. And the second outcome is the change of Western Ontarioand McMaster Universities Osteoarthritis Index (WOMAC) total score from baseline to 4 weeks of KOA patients.

### 2.10 Adverse reactions

Serious adverse reactions (SARS) and suspected unexpected serious adverse reactions (SUSARS) will be registered according to the protocol approved by the Chinese Medicine Agency (CMA).

## 3 Discussion

In this study we choose KOA as the vehicle for studying the phenomenon of acupoint sensitization. Because first, KOA has some clear pathological reaction points on the body surface often represented by changes in pain and thermo sensitivity.^[14]^ Secondly, KOA is the preponderant illness for acupuncture therapy.^[32]^

Von Frey monofilament is a classic pain measurement method in patients with allodynia and neuropathic pain.^[30,33]^ Clinical trials have shown that the Electronic Von Frey method is reliable for measuring MPTs. Electronic Von Frey measurement is based on an increasing stimulus intensity, Suzuki et al proved the electronic Von Frey device were significantly higher and more consistent than pinprick stimulators when tested MPTs in human.^[34,35]^

Micro dialysis is an in vivo sampling technique implant a probe into a dialysis site, such as brain tissue, muscle tissue, blood vessels, then to dialysis a biological sample.^[36]^ This method reflects real-time dynamic response to the components of tissue fluid and has been widely used in clinical practice since its beginning.^[37]^ Takahiro Takano applied micro dialysis technology to ST36 (Zusanli) Point to analyze the adenosine concentration of local tissue fluid after local acupuncture.^[38]^ In 2017, Swedish researchers placed micro dialysis probe into the masticatory muscles to analyze the changes of inflammatory molecular components in patients with masticatory cramps.^[39]^ The above studies found that the application of micro dialysis technology is mature and safe in clinical practice. In this study, we will use the micro dialysis device provide by CMA company. And the CMA 66 Liner Catheter has a semi permeable membrane with 20,000 Dalton molecular weight cutoff only small-molecular substances can pass through the membrane while micro molecular substances, such as proteins and cells are excluded.^[40]^ Considering the amount of substances exchange at micro liter level (0.3 μl/ minute), disruption of the fluid balance and the metabolic process could be neglected.^[41]^

Metabolomics is a comprehensive approach to the evaluation of small molecules involved in the qualitative and quantitative analysis of total metabolites in biological samples. It has been reported to have a major impact on physiological studies, disease diagnosis, biomarker discovery, and the search for interference pathways associated with disease or treatment.^[42]^ Changes in the state of the organism from a healthy state to a disease state are the overall result of fluctuations in organ metabolism, and the types and concentrations of metabolites are constantly changing. The metabolome is closer to representing the phenotype, which is the final product and is the most stable at the genome, transcriptome, and proteome levels, reflecting cellular metabolism.^[42]^ One of the most important goals of metabolomics research is to find specific and sensitized biomarkers that can clearly detect disease, and seems to be an effective way to understand disease-related phenotypic changes.^[43]^

LC-MS/MS method for analysis metabolomics has been used in various kinds of diseases, such as intestinal, aging and cardiovascular disease,^[44]^ colonitis,^[45]^ and rheumatoid arthritis.^[46]^ It is been developed to require substantially less starting volume (25 μl) method, also very sensitized and amenable to high-throughput analysis,^[47]^ the less volume requirement is very consistent with the collection of human micro dialysis sampling in our study design.

Studies have confirmed that acupuncture operations on sensitized acupoints can help to improve the clinical efficacy of acupuncture.^[16,17]^ We reckon that acupuncture may cause changes of small molecules substances in sensitized acupoints. These finding may also attribute to clinical appliance.^[14–16]^ Although there were studies reported that local mast cell degranulation might be the crucial progress to sensitization in the KOA animal model,^[48–51]^ it still remains unclear whether the rest of the substances are involved in this process and their role in acupuncture treatment process. Therefore, in this study, we will use LC-MS/MS to analyze the dialysate, and then apply non-targeted metabolomics strategy based on high-throughput method with huge data processing platform to describe the metabolic profiling of dialysate from sensitized acupoints of KOA patients. With these methods we hope to find other potential key substances of sensitized acupoints in KOA patients. This study may be able to provide a basis for understanding the systemic biological mechanisms of acupoints sensitization.

There are several limitations of this study. First, the sample size is small. But the results of this study may help us to know about some crucial information of sensitized acupoints in KOA patients. Other potential limitation of this study is the sensitization phenomenon has many types, such as pain-sensitized, heat-sensitized, morphologic change, and so on.^[21]^ We only study pain-sensitized phenomenon of KOA patient, the results of this study will be limited use in this disease to explain pain-sensitized.

## Author contributions

LS, CXN contributed equally to this work. LS, CXN wrote the study protocol and drafted this manuscript, YHY and YSG made a substantial contribution to the study protocol design.THJ, ZCY, and LP participated in the qualitative study design and in the critical revision. LLZ and YHY participated in the critical revision of the manuscript. TY helped to draft the manuscript. YHY and YSG had final responsibility for the decision to submit for publication. All authors read and approved the final manuscript.

Sheng Lee orcid: 0000-0002-9534-5751.
